# Beyond the Prognostic Value of 2-[^18^F]FDG PET/CT in Prostate Cancer: A Case Series and Literature Review Focusing on the Diagnostic Value and Impact on Patient Management

**DOI:** 10.3390/diagnostics12030581

**Published:** 2022-02-24

**Authors:** Roberto Borea, Diletta Favero, Alberto Miceli, Maria Isabella Donegani, Stefano Raffa, Annalice Gandini, Malvina Cremante, Cecilia Marini, Gianmario Sambuceti, Elisa Zanardi, Silvia Morbelli, Giuseppe Fornarini, Sara Elena Rebuzzi, Matteo Bauckneht

**Affiliations:** 1 Medical Oncology Unit 1, IRCCS Ospedale Policlinico San Martino, 16132 Genova, Italy; 2 Department of Internal Medicine and Medical Specialties (Di.M.I.), University of Genova, 16132 Genova, Italy; 3 Medical Oncology Unit 2, IRCCS Ospedale Policlinico San Martino, 16132 Genova, Italy; 4 Department of Health Sciences (DISSAL), University of Genova, 16132 Genova, Italy; 5 Nuclear Medicine, IRCCS Ospedale Policlinico San Martino, 16132 Genova, Italy; 6 CNR Institute of Molecular Bioimaging and Physiology (IBFM), 20054 Segrate, Italy; 7 Academic Unit of Medical Oncology, IRCCS Ospedale Policlinico San Martino, 16132 Genova, Italy; 8 Medical Oncology Unit, Ospedale San Paolo, 17100 Savona, Italy

**Keywords:** prostate cancer, positron emission tomography, 18F-Fluorodeoxyglucose, diagnosis, staging, treatment response assessment

## Abstract

The role of 2-deoxy-2-[^18^F]fluoro-D-glucose Positron Emission Tomography/Computed Tomography (FDG PET/CT) in the management of prostate cancer (PCa) patients is increasingly recognised. However, its clinical role is still controversial. Many published studies showed that FDG PET/CT might have a prognostic value in the metastatic castration-resistant phase of the disease, but its role in other settings of PCa and, more importantly, its impact on final clinical management remains to be further investigated. We describe a series of six representative clinical cases of PCa in different clinical settings, but all characterised by a measurable clinical impact of FDG PET/CT on the patients’ management. Starting from their clinical history, we report a concise narrative literature review on the advantages and limitations of FDG PET/CT beyond its prognostic value in PCa. What emerges is that in selected cases, this imaging technique may represent a useful tool in managing PCa patients. However, in the absence of dedicated studies to define the optimal clinical setting of its application, no standard recommendations on its use in PCa patients can be made.

## 1. Introduction

Prostate cancer (PCa) is the most common malignancy and the second most frequent cause of cancer-related death among the male population in Western Countries [1]. According to the clinical history, most PCa patients present with locoregional disease. Only a tiny percentage of them are likely to metastasise over time, while a minority of PCa patients present with metastatic disease at diagnosis [2]. Moreover, the clinical behaviour of PCa is widely heterogeneous, ranging from an indolent and hormone-responsive disease to a highly aggressive and treatment-resistant one [2,3]. As a reflection, clinical management may range from active surveillance in low-risk tumours to definitive treatment (radiation therapy/radical prostatectomy) in locoregional ones, up to systemic therapies for patients with advanced disease [2,3].

Due to this wide range of biological behaviour, accurate diagnostic tools for disease diagnosis and staging and assessing tumour aggressiveness is fundamental to support clinical decisions and improve PCa patients’ management.

Standard staging and risk assessment of PCa patients include multiparametric magnetic resonance imaging (mpMRI) before prostate biopsy, thoracoabdominal computed tomography (CT), and bone scan for staging intermediate- or high-risk patients [2,4]. Currently, other imaging techniques are not routinely recommended by any international guideline [2]. However, a recent prospective randomised phase III study showed that Positron Emission Tomography (PET) with [^68^Ga]Ga-Prostate-Specific Membrane Antigen-11 (PSMA) provided superior accuracy than conventional imaging (CT and bone scan) [5]. Similarly, PSMA PET imaging is increasingly replacing conventional imaging based on its superior sensitivity and specificity for the restaging of PCa patients with biochemical recurrence [2,6,7]. Moreover, other PET tracers have been used in the restaging setting, including choline labelled with either [^11^C]- or [^18^F]- [8] and [^18^F]-Fluciclovine [9].

**Figure 1:**
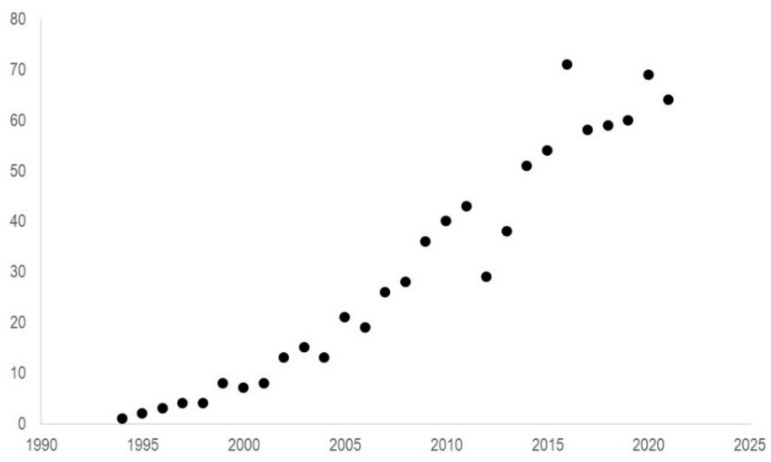
The number of papers (Y-axis) available in the PubMed database published in each year since 1994 (X-axis) containing the terms “Fluorodeoxyglucose” and “prostate”.

Fluorodeoxyglucose [^18^F] (FDG) PET/CT is one of the most used imaging techniques in oncology: it is used in the diagnosis, staging, prognosis prediction, monitoring of treatment response, and surveillance of various cancer types. However, literature data about its role in the management of PCa patients is still controversial [10,11]. Besides initial studies claiming its limited value in this cancer type because of the generally low glucose uptake of PCa cells [12,13], in the last years, a large amount of data has been published on the role of FDG PET imaging in PCa (Figure 1).

Among them, a large part of the published studies showed that FDG imaging might be useful mainly because of its prognostic value in the metastatic castration-resistant phase of the disease (mCRPC) [11,14,15,16]. By contrast, the role of FDG PET in other settings of PCa and the comparison with conventional imaging remains to be further investigated. Starting from the clinical history of emblematic clinical cases, we report a concise narrative literature review on the potential diagnostic uses of FDG PET in PCa, beyond its prognostic value.

## 2. FDG PET in the Incidental Detection of the Primary PCa

**Figure 2:**
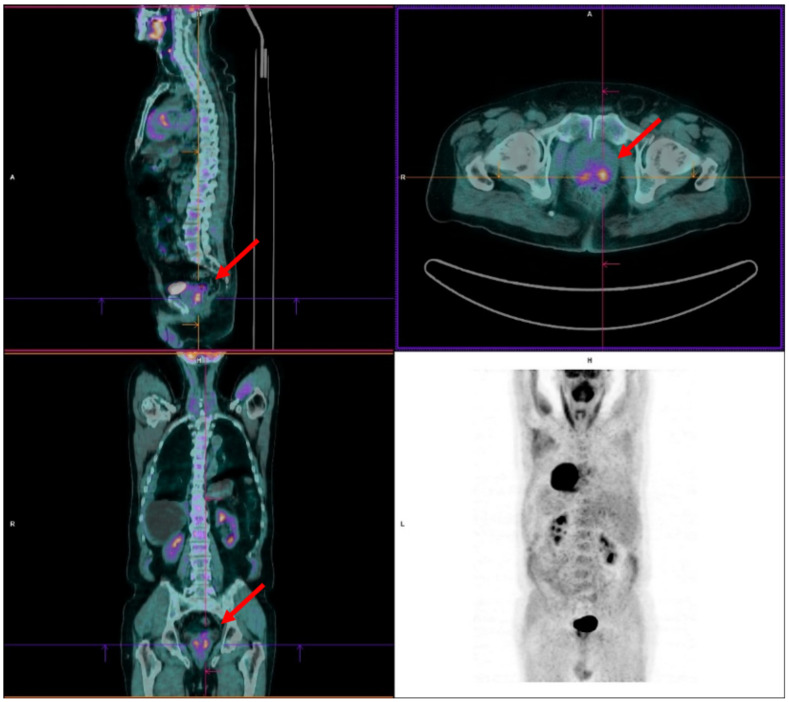
Incidentally increased tracer uptake in two areas (red arrows) of the posterior lobe of the prostate gland resulting in PCa at histopathology.

A 76-year-old male with a clinical history of asbestos exposure presented to our hospital for recurrent coughing and dyspnoea in January 2020. The physical examination revealed inaudible breath sounds and dullness at percussion in the right lung, and the chest X-ray showed the presence of right pleural effusion. The patient underwent a thoracentesis with three litres of clear pleural fluid evacuation. The cytological analysis revealed pleural lymphocytosis with some mesothelial cells but no evidence of atypical cells. In February 2020, a chest CT scan confirmed right pleural effusion and pneumothorax, with no evidence of suspected pulmonary lesions. A subsequent FDG PET was performed to guide the diagnosis to identify occult lung or pleural proliferative lesions. As no radiopharmaceutical uptake was shown in the mediastinum, the pleural effusion was considered related to the pneumoconiosis. However, increased tracer uptake was observed in two areas of the posterior lobe of the prostate gland, with the maximum standardised uptake value (SUV_max_) on the left posterior prostatic lesion equal to 5.6 (Figure 2).

The specific prostate antigen (PSA) level result slightly increased (5 ng/mL, with the upper limit normal of 2.5 ng/mL). The subsequent urological evaluation confirmed the presence of a left apical prostatic nodule. In the same month, transrectal ultrasound (TRUS)-guided prostatic biopsies were performed, and the histological examination confirmed the diagnosis of PCa with a Gleason score (GS) of 6 (3 + 3) in all taken cores. Given the low risk of the disease, the patient started active surveillance.

**Figure 3:**
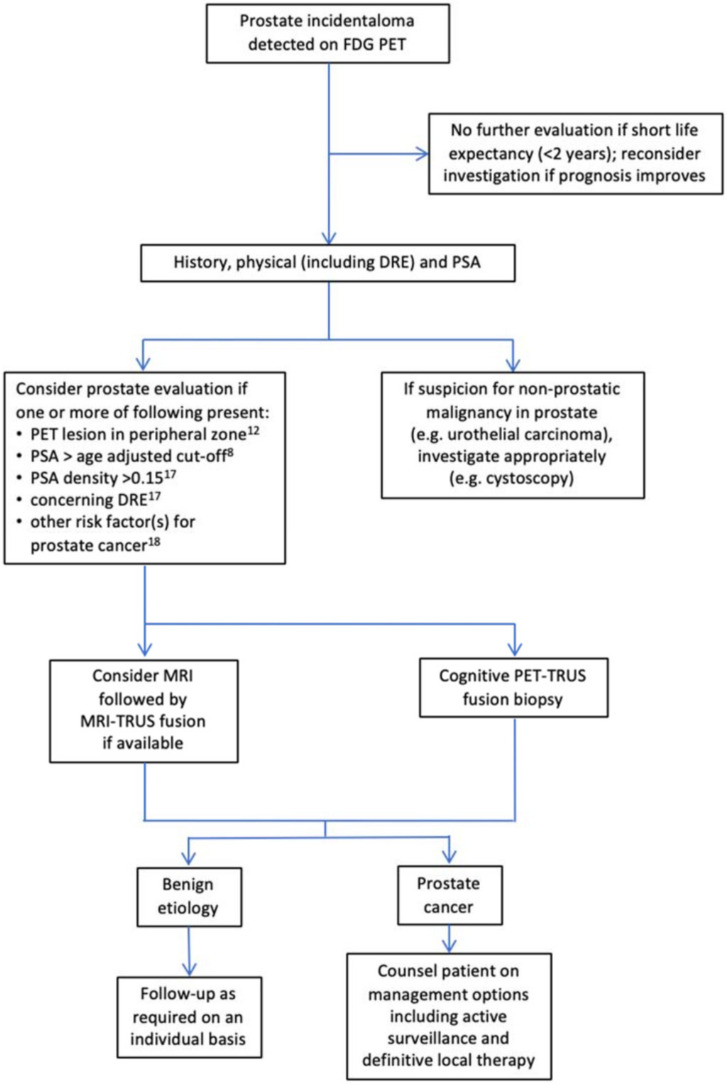
Tentative diagnostic algorithm to investigate prostate incidentalomas detected on FDG PET/CT. Adapted with permission from Mannas et al. [23]. *DRE*—digital rectal exam; *MRI*—magnetic resonance imaging; *PSA*—prostate-specific antigen; *TRUS*—transrectal ultrasound.

In this clinical case, FDG PET led to the accidental finding of a primary PCa. Despite FDG PET having little utility for detecting primary PCa due to the low sensitivity [17,18,19] and specificity [20,21], the incidental finding of focal tracer uptake within the prostate gland when FDG PET is performed for unrelated conditions represents a distinct clinical scenario. A few studies, including a systematic review and meta-analysis, assessed the prevalence and risk of an incidental neoplastic FDG PET uptake in the prostate gland [22,23]. FDG prostatic incidentaloma is a relatively rare event, ranging from 1 to 1.8% [22,23]. According to the study by Bertagna and colleagues, in patients screened for PCa after the incidental finding of focal prostatic FDG uptake, the prevalence of malignancy is 12.5% [22]. Thus, although FDG PET is not necessarily diagnostic for the PCa primary detection when focal intense radiotracer uptake is seen, it should not be ignored and should lead to diagnostic assessment. While a validated flowchart for prostatic FDG-avid incidentalomas is not included in the current international guidelines, Mannas et al. have recently proposed a tentative diagnostic algorithm (Figure 3) [23].

The prognostic value of FDG imaging also applies to this setting. Indeed, in a study conducted on 42 patients with primary PCa submitted to FDG imaging before radical prostatectomy, patients with higher SUV_max_ of the primary PCa showed worse long-term survival [24]. However, it should be noted that in our clinical case, the intense focal FDG uptake was associated with a low-risk primary PCa. On the other hand, given the low sensitivity of FDG PET in this clinical setting, low-FDG-avid high-grade PCa primary tumours can also be eventually observed [25]. Indeed, while it is theoretically presumable to observe increased tracer uptake in high-grade tumours [13,26,27,28], in some instances, this correlation can be scarcely reproduced at a single patient level in the real-world setting. This is consistent with the study by Mannas et al., in which about 18% of the prostate FDG-avid incidentalomas were low-risk PCa [23]. Therefore, the focal FDG uptake incidental finding should always warrant attention, regardless of the SUV_max_ value.

## 3. FDG PET as a Potential Tool for Therapy Monitoring in Hormone-Sensitive PCa

A 76-year-old man was routinely submitted to FDG PET in our hospital for treatment assessment for gastric cancer. He received radical surgery in March 2017 and adjuvant chemotherapy from May to August 2017. The first post adjuvant treatment FDG PET was performed in October 2017 and showed two low-FDG-avid lesions (pyloric site and left adrenal gland), which were not defined as clearly active disease sites. Therefore, according to the inconclusive finding, strict imaging monitoring was agreed with the patient.

**Figure 4:**
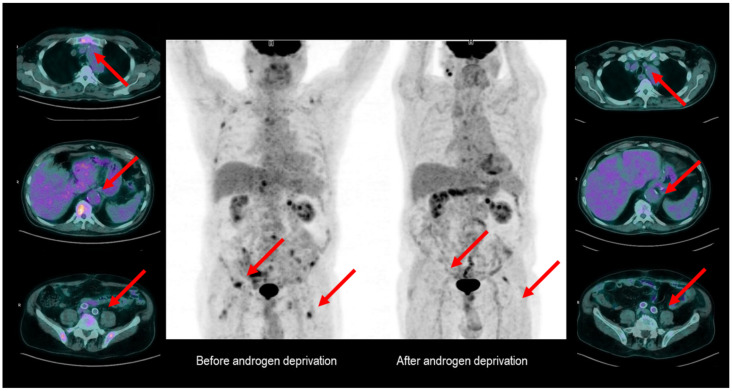
Androgen deprivation-induced metabolic response in hormone-sensitive PCa. The red arrows indicate metabolically active lesions.

The following FDG PET of March 2018 showed an FDG uptake’s metabolic stability of the left adrenal gland and the disappearance of the gastric metabolic uptake. The same PET scan also revealed several FDG-avid bone lesions involving the sacrum, the left scapula, some right ribs, the sternum, some vertebras, the pelvis, and both femurs, all attributable to the oncological disease. The bone lesions had an osteoblastic nature and low FDG avidity. A serum PSA evaluation was subsequently performed, which yielded abnormal results (51 ng/mL). The radiological characteristics of the bone lesions, together with the evidence of increased PSA, raised the suspicion of the presence of a metastatic PCa. On this basis, the patient started androgen deprivation therapy (ADT) with triptorelin 11.25 mg every three months. The diagnosis of PCa was confirmed *ex adiuvantibus* by dropping the PSA levels to 0.9 ng/mL after only three months of ADT. Interestingly, the subsequent FDG PET performed for the gastric cancer restaging (August 2020) detected a “metabolic shutdown” of the previously described FDG-avid bone lesions (Figure 4), which was consistent with the dropping the PSA levels.

In the present case, FDG PET allowed for identifying bone metastases from an occult PCa. It displayed the metabolic regression of metastatic lesions under ADT when repeated for unrelated reasons.

This finding is in line with preclinical studies, showing that FDG uptake by hormone-sensitive PCa declines with androgen withdrawal and increases once the castration resistance occurs [29]. In the clinical setting, Oyama et al., showed that following ADT, the greater is the reduction in FDG uptake, the deeper is the reduction in serum PSA [30]. This phenomenon involved the primary lesion and the metastatic sites, suggesting that the glucose utilisation by tumour cells is systematically suppressed by androgen ablation. Comparable findings were reported by Jadvar and colleagues [31,32]. The likeliest explanation for these results is that glucose transporter 1 (GLUT-1) is downregulated in androgen receptor (AR)-dependent cells, representing a signature of PCa hormone responsiveness [33,34].

On the other hand, the metabolic change induced by the hormone deprivation might be relatively independent of the antiproliferative effect of androgen deprivation itself, as preclinical data showed that the SUV could remain unchanged after chemotherapy [35]. Fox et al. analysed 133 mCRPC patients through a dual tracer PET/CT approach (FDG and 18F-Fluorodihydrotestosterone), identifying at least four different phenotypes of metastatic lesions based on a dichotomous classification of imaging findings [36]. Using histopathology as a reference, the authors observed that when mCRPC lesions were FDG-avid, they were less differentiated and low expressing AR [36]. Moreover, the higher was the preponderance of the FDG+/AR− disease, the poorer was the clinical outcome [36].

Based on these considerations, FDG PET could be used (at least in specific cases, such as the non-PSA secreting PCa) in the imaging evaluation of response to ADT in hormone-sensitive PCa and for the early prediction of hormone refractoriness.

## 4. FDG PET as a Tool to Improve Systemic Treatment Selection in the Castration-Resistant Phase of the Disease

Two emblematic clinical cases will be discussed in this paragraph.

### 4.1. Case 1

A 79-year-old man with a medical history of dyslipidaemia, type 2 diabetes mellitus, and heart failure was diagnosed with PCa GS of 9 (4 + 5) in 2015. The PSA at the time of diagnosis was 9.7 ng/mL. Preoperative staging imaging (bone scintigraphy and CT scan) did not reveal distant metastasis. Radical prostatectomy was performed with the histological result of pT3b pN1 cM0. Due to positive surgical margins and the presence of lymph node metastasis, subsequent adjuvant hormone therapy was prescribed.

After the PSA increased during ADT, Fluorocholine (^18^F) (choline) PET/CT was performed in January 2018, showing mediastinal centimetric lymphadenopathies. The patient started first-line therapy of mCRPC with Abiraterone Acetate. After a few months, a new biochemical disease progression was found. Therefore, the patient was studied with an 18F-Choline which highlighted the appearance of bone metastases at the cervical, dorsal, and lumbar spine. These findings were confirmed by a full spine MRI examination. Hence, in November 2018, the patient started chemotherapy with Docetaxel, and external beam radiotherapy on spine skeletal localisations was performed. PSA prechemotherapy was 34 ng/mL and, during chemotherapy, reached a nadir of 15 ng/mL in January 2019. The last administration of Docetaxel was in April 2019, with an increased PSA of 19.4 ng/mL. Given the biochemical progression during chemotherapy, the extent and the symptomatology of the skeletal disease, (^223^Ra)RaCl_2_ (Ra-223) therapy was proposed.

**Figure 5:**
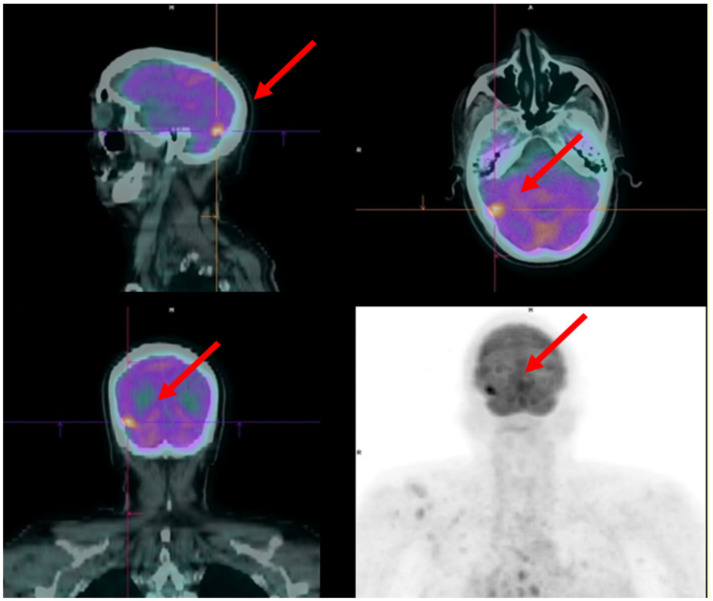
Brain metastasis in the right temporo-basal cortex (red arrows) incidentally discovered by 18F-FDG PET in an mCRPC patient candidate to the Ra-223 therapy.

Bone scintigraphy and a CT scan were performed as required by guidelines to restage the patient, showing multiple pathological skeletal findings in the absence of visceral metastases. A baseline FDG PET was also performed as part of a research protocol of our institution [37,38,39,40]. The exam showed multiple bone metastases and an area of relatively increased tracer uptake in the brain between the temporal and right occipital cortex (Figure 5). Theoretically, this finding might be related to malignancy other than prostate cancer. However, prostate cancer localisation was considered highly suspicious given the clinical context. A brain MRI was performed confirming the presence of an expansive lesion of about 15 × 13 mm in the right temporo-basal cortical-subcortical supratentorial region with inhomogeneous contrast enhancement and discrete surrounding oedema. In the subtentorial region, at the left posterior hemispheric cerebellar cortical level, another small lesion of about 5 mm and similar characteristics was also detected. Identifying cerebral localisations of the disease made it necessary to change the therapeutic strategy. After stereotaxic brain radiotherapy, the patient started a new line of chemotherapy with Cabazitaxel.

### 4.2. Case 2

**Figure 6:**
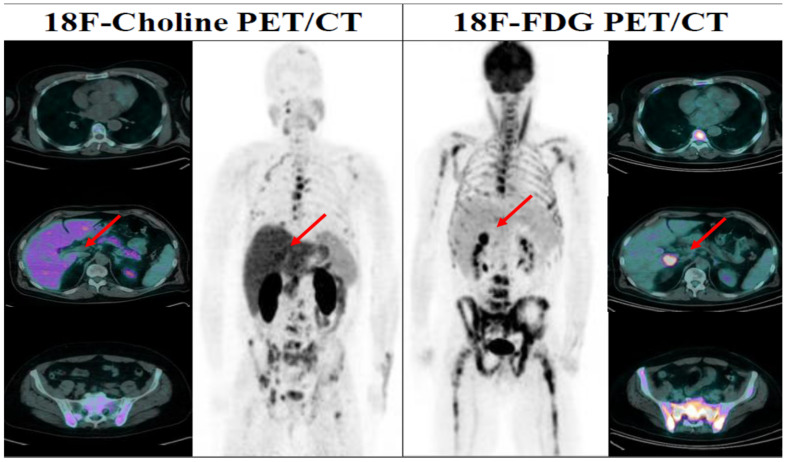
Comparison of 18F-Choline PET/CT and ^18^F-FDG PET; 18F-FDG PET shows high osteo-medullary tracer uptake and a focal area of uptake in the liver (red arrows), while 18F-Choline PET shows only a few bones metastasis.

In February 2017, a 58-year-old man was diagnosed with PCa and bone metastases from the beginning. Due to the high volume of disease, the patient received first-line therapy with Docetaxel for six cycles as the CHAARTED protocol [41] with a good biochemical response (PSA: from 249 to 0.15 ng/mL). About six months after the end of the chemotherapy, the PSA started to rise again, and second-line therapy with cabazitaxel was started, but with a poor clinical and biochemical response. The patient received then a third-line treatment with enzalutamide but again without clinical and biochemical benefits. At this time point, a choline-PET/CT and a bone scan were performed with the evidence of progressive disease but still localised to the bones. Due to the bone-limited disease, Ra-223 was considered a suitable therapeutic opportunity. All the selection criteria according to conventional imaging were respected at that moment, but an FDG PET was performed as part of a research protocol of our institution [37,38,39,40]. Surprisingly, the FDG PET scan revealed a visceral metastasis at the sixth hepatic segment (SUVmax 12.3), thus excluding the patient to the Ra-223 treatment (Figure 6).

These two clinical cases show how FDG PET findings may impact the therapeutic management of mCRPC patients by contraindicating systemic therapies that would have had partial clinical benefits. The therapeutic scenario of advanced PCa radically changed in the last years due to the improved knowledge of PCa biology and progression mechanisms. New molecules have been registered for mCRPC, and several emerging compounds are currently in the path of their validation. As combinations of these drugs have not always been associated with positive results [42,43], the sequential strategy remains the dominant therapeutic approach in clinical practice. Subgroup analyses showed that optimising treatment sequencing may favourably impact the clinical outcome of mCRPC patients [44,45,46,47,48]. However, choosing the right drug at the right time is challenging at a single patient level. In this scenario, there is an urgent need to identify reliable biomarkers that may early and noninvasively estimate treatment efficacy before administration, potentially improving the best drug’s selection to the right patient.

In a recent retrospective study by our group, FDG PET has been used to estimate the systemic treatment response in mCRPC receiving chemotherapy or androgen receptor-targeted therapies [16]. We observed that FDG PET imaging might identify subgroups of patients most likely to benefit from a specific treatment, thus optimising treatment sequencing. Although not systematically [49], lower FDG uptake is generally associated with higher success rates for Androgen-Receptor Targeted Agents. The present cases extend these previous observations, suggesting that the favourable impact of FDG PET might also be related to the more accurate staging of the extent of the disease.

On the other hand, in the last years, FDG PET has been increasingly included in clinical trials testing PSMA radioligand therapy in the mCRPC setting. Indeed, a crucial selection criterion for PSMA radioligand therapy is the homogeneous expression of PSMA in tumour sites, as assessed by PSMA PET/CT [50]. In this scenario, additional FDG PET/CT appears to help detect more aggressive disease [14,15,16] and predict and optimise response rates to radioligand therapy [51]. Thus, FDG PET can be a useful complementary imaging tool to guide the choice or the exclusion of systemic therapy in mCRPC patients.

## 5. FDG PET as a Potential Tool for Therapy Monitoring in the Castration-Resistant Phase of the Disease

**Figure 7:**
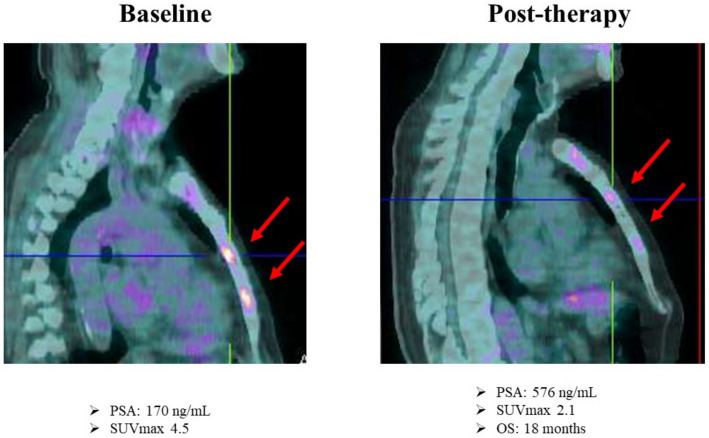
18F-FDG PET at baseline and after three cycles of Ra-223. The red arrows indicate metabolically active bone lesions.

In July 2014, an 80-year-old man with anamnesis of chronic ischemic heart disease presented to the medical attention for pollakiuria and underwent a PSA measurement which showed an increased PSA level (6.86 mcg/L). A month later, a TRUS was performed, showing an enlarged prostate gland with no evident prostatic nodules. Subsequently, the patient underwent transurethral resection of the prostate (TURP), and the histological examination revealed the diagnosis of PCa with a GS of 7 (4 + 3). In October 2014, considering the age and the comorbidities, after negative CT and bone scan, the patient started ADT. After about four years, in May 2018, PSA was slowly increasing (1.6 mcg/L) compared with previous months, so an 11C-choline PET was performed for restaging and showed the presence of multiple and diffuse bone metastases. Given the mCRPC status, the patient received Abiraterone Acetate for 11 months until June 2019. In this month, due to a new increase of PSA (6.2 mcg/L), a bone scan was performed showing progressive disease with an increased number of bone lesions. In August 2019, considering the limited bone progression, the patient started Ra-223 therapy. Before therapy administration, the patient performed an FDG PET as part of a research protocol [37,38,39,40]. The PET scan confirmed the presence of increased radiopharmaceutical uptake in the right orbital cavity (SUV_max_ = 10), right scapula, multiple vertebral bodies (SUV_max_ = 10), sacrum, and right femoral neck (SUV_max_ = 5). In November 2019, after three Ra-223 administrations, a new biochemical progression was documented (PSA 15 mcg/L). The bone scan was repeated and showed a diffuse increased uptake of the previously described bone lesions. FDG PET was then performed to improve the differential diagnosis between flare-up effect induced by Ra-223 therapy and disease progression. Intriguingly, it showed a decreased radiopharmaceutical uptake of all bone lesions (SUV_max_ = 3.3 vs. 10 of the right orbital cavity, SUV_max_ = 5.8 vs. 10 of vertebral bodies) (Figure 7). Considering the FDG PET documented metabolic response, Ra-223 was continued until the fifth cycle, when a clinical and biochemical progression was observed in January 2020. In May 2020, the patient started Enzalutamide until therapy discontinuation for toxicity and then died six months later.

Although the efficacy of Ra-223 was established in a randomised placebo-controlled trial in mCRPC patients, response to this treatment at the single patient level is still difficult to evaluate [52]. PSA responses are rare, and response assessment with conventional imaging (CT and bone scan) can easily establish the disease progression of the bone metastases, but no disease response. The evaluation of response using CT in patients with predominantly bone metastases is challenging, as bone metastases are not considered in RECIST response criteria for clinical trial purposes [53]. On the other hand, the scintigraphic “flare phenomenon”, which is the apparent scintigraphic progression associated with clinical response [54], reduces the bone scan specificity.

In this scenario, alternative biochemical and imaging approaches are needed for treatment response evaluation. PET imaging with different radiotracers, including 18F-NaF, choline, and PSMA, has been tested in this field [55,56,57,58]. In a retrospective study conducted by our group, the decrease in Metabolic Tumor Volume measured by 18F-FDG PET leads to a better long-term OS in patients treated with Ra-223 [37,38]. The present clinical case represents an emblematic example of this finding, as FDG PET identified a flare effect rather than progressive disease despite biochemical and bone scan progression. In conclusion, in selected cases, FDG PET may help correctly assess the treatment response in the mCRPC disease, unmasking possible flare-up which could be misinterpreted as progressive disease. Of note, the usefulness of FDG imaging in the response assessment has also been shown in mCRPC treated with chemotherapy [59].

## 6. Conclusions

As the above cases exemplify, FDG PET may be a useful tool in managing patients with PCa at different time points of clinical history such as diagnosis, staging, systemic treatment selection, and evaluation of response.

Although FDG PET has little utility in diagnosing primary PCa, the finding of prostate incidentalomas on FDG imaging should always lead to diagnostic assessment, regardless of the SUVmax value. Concerning treatment monitoring, FDG PET can be a useful tool to assess the metabolic response to ADT and for the early prediction of hormone refractoriness in hormone-sensitive PCa. FDG PET may also assess the treatment response in the mCRPC disease, identifying potential flare-ups which could be misinterpreted as progressive disease. Generally, FDG PET can help physicians select the best systemic treatment, thus optimising treatment sequencing and correctly assessing the treatment response when the results of conventional imaging are indeterminate or of limited utility.

We suggest that FDG PET should be considered in select clinical scenarios from diagnosis to treatment monitoring of PCa. However, until additional studies are conducted to decipher the optimal clinical setting of its application, no standard recommendation can be made.

## Data Availability

Not applicable.
